# The Role of Molecular Modeling in TiO_2_ Photocatalysis

**DOI:** 10.3390/molecules22040556

**Published:** 2017-03-30

**Authors:** Zekiye Cinar

**Affiliations:** Department of Chemistry, Yildiz Technical University, 34220 Istanbul, Turkey; cinarz@yildiz.edu.tr; Tel.: +90-212-383-4179

**Keywords:** TiO_2_, photocatalysis, DFT, quantum mechanics, molecular modeling

## Abstract

Molecular Modeling methods play a very important role in TiO_2_ photocatalysis. Recent advances in TiO_2_ photocatalysis have produced a number of interesting surface phenomena, reaction products, and various novel visible light active photocatalysts with improved properties. Quantum mechanical calculations appear promising as a means of describing the mechanisms and the product distributions of the photocatalytic degradation reactions of organic pollutants in both gas and aqueous phases. Since quantum mechanical methods utilize the principles of particle physics, their use may be extended to the design of new photocatalysts. This review introduces molecular modeling methods briefly and emphasizes the use of these methods in TiO_2_ photocatalysis. The methods used for obtaining information about the degradabilities of the pollutant molecules, predicting reaction mechanisms, and evaluating the roles of the dopants and surface modifiers are explained.

## 1. Introduction

Molecular modeling is the art of representing molecular structures mathematically and simulating their behavior with quantum mechanical methods. Quantum mechanical methods allow us to study chemical phenomena by running calculations on computers rather than carrying out experiments. Geometries and properties of the transition states, excited states or other short-lived species can only be calculated by using quantum mechanical methods. They utilize the principles of particle physics and examine structure as a function of electron distribution. Therefore, they can be used to design new photocatalysts, to even analyze reactions which have not yet been carried out. Quantum mechanical methods generate data on geometries (bond lengths, bond angles, and torsion angles), energies (total energies, heats of formation, activation energies, and thermodynamic properties), electronic (frontier orbital energies, charge distributions, and dipole moments) and spectroscopic properties (absorption thresholds, vibrational frequencies, and chemical shifts).

TiO_2_ photocatalysis is an advanced oxidation process to destroy hazardous compounds in water or air [1,2,3,4]. The process is non-energy intensive, operates at ambient conditions and able to mineralize organic pollutants using only atmospheric oxygen as the additional chemical species. Because of its low cost, long-time stability, chemical inertness and high activity, and TiO_2_ has been proven to be the most effective photocatalyst for this process [5,6,7,8]. The photocatalytic reactions on TiO_2_ are initiated by band-gap excitation and subsequent generation of electron–hole (e^−^/h^+^) pairs that can initiate redox reactions on the surface. Electrons are trapped at surface defect sites (Ti^3+^) and removed by reactions with adsorbed molecular O_2_ to produce superoxide anion radical O_2_•^−^, while holes react with adsorbed water molecules or OH^−^ ions to produce •OH radicals. •OH radicals are considered to be the principal reactive species responsible for the degradation reactions. However, TiO_2_ has a wide band-gap (~3.2 eV) and is only excited by UV-light; it is inactive under visible light irradiation. This feature of TiO_2_ inhibits the utilization of solar energy as a sustainable energy source for its excitation because only 5% of the incoming solar energy on the earth’s surface is in the UV range. Besides, electron–hole recombination speed is too fast to allow any chemical reaction, due to short charge separation distances within the particle. The majority of the e^−^/h^+^ pairs generated upon band-gap excitation are lost through recombination instead of being involved in redox processes at the surface. The e^−^/h^+^ recombination process not only decreases the quantum yield but also decreases the oxidation capability of TiO_2_ [8,9]. Therefore, in recent years, in order to utilize sunlight instead of UV irradiation, studies have begun to develop the next generation of TiO_2_, well-tailored photocatalysts with high photocatalytic activities under visible light irradiation. One way to achieve this is doping of impurities into the TiO_2_ matrix in order to reduce the band-gap. The methods used are transition metal doping [10,11,12,13,14,15,16], metal-ion implanting, surface modification, non-metal doping [17,18,19,20,21,22,23,24,25,26,27,28,29] and cooping [30,31,32,33,34,35,36,37,38]. However, each method has shown both positive and negative effects.

The common positive effect of doping and surface modification of TiO_2_ is that the absorption edge shifts to the red region of the spectrum and the photocatalytic activity increases, but, the key question to be answered is how doping or surface modification achieves this. On the other hand, photocatalytic degradation reactions of organic contaminants may take place through the formation of harmful intermediates that are more toxic than the original compound. In order to eliminate certain reaction paths yielding such hazardous compounds, the mechanisms and the nature of the reactions should be known. As for the mixtures of different pollutants, the reactivities of the individual molecules are also needed. All of these problems can be solved by computational techniques based on the principles of quantum mechanics. The aim of this review is to introduce molecular modeling methods briefly and emphasize the use of these methods in TiO_2_ photocatalysis. The methods used for obtaining information about the degradabilities of the pollutant molecules, predicting reaction mechanisms and evaluating the roles of the dopants and surface modifiers are explained.

## 2. Molecular Modeling Methods

Molecular modeling studies start with generating a model of the molecule under investigation. Models are generated in the computer by defining the relative positions of the atoms in space by a set of Cartesian coordinates. A reasonable and reliable starting geometry essentially determines the quality of the calculations. There are mainly four classes of molecular modeling methods; molecular mechanics (MM), electronic structure, post-ab initio and molecular dynamics methods [39,40,41]. All molecular modeling methods compute the energy and related properties of a particular molecular structure. However, the generated model of a given molecule does not have ideal geometry; therefore, a geometry optimization must be performed subsequently. Geometry optimizations locate the lowest energy molecular structure in close proximity to the specified starting structure. Geometry optimizations depend primarily on the gradient of the energy which is the first derivative of the energy with respect to atomic positions. Gradient is the force acting on the structure. The lowest energy structure obtained through energy-minimization techniques corresponds to the geometry with zero gradient. Vibrational frequencies of molecules resulting from interatomic motions within the molecule may also be calculated by molecular modeling methods. Frequencies depend upon the second derivative of the energy with respect to atomic structure and they can be used to predict thermodynamic properties of the molecule.

### 2.1. Molecular Mechanics Methods

Molecular mechanics (MM) methods use the laws of classical physics and a set of experimental data for atom types. MM methods do not treat electrons; instead, they perform computations based upon the interactions among the nuclei. This approximation makes MM calculations quite inexpensive so they can be used for very large systems.

### 2.2. Electronic Structure Methods

Electronic structure methods use the laws of quantum mechanics rather than classical physics. The fundamental equation of quantum mechanics is the Schrödinger equation;

**HΨ** = E**Ψ** (1)

where E is the energy of the system, **Ψ** is the wavefunction which defines Cartesian and spin coordinates of atoms, and **H** is the Hamiltonian operator including kinetic and potential energy terms. However, exact solutions to the Schrödinger equation are not computationally practical. Approximations must be introduced in order to apply the method to multi-electronic and polyatomic systems. Electronic structure methods are characterized by their various mathematical approximations to the solution of the Schrödinger equation.

“*Semi-empirical methods*” use experimental data to simplify the computation and solve an approximate form of the Schrödinger equation ignoring complex differentials. They reduce the computational cost by reducing the number of complex integrals. “*Ab initio*” methods use no experimental parameters. Instead, they use the well-known physical constants, and solve the equation directly using only the principles of quantum mechanics.

Semi-empirical methods are relatively inexpensive and provide reasonable qualitative descriptions of molecular systems. In contrast, ab initio methods provide accurate quantitative predictions of energies and structures. Both semi-empirical and ab initio methods depend upon Hartree–Fock (HF) theory. In HF theory, single-electron wavefunctions are used. The one-electron wavefunctions are molecular orbitals which are given as a product of a spatial orbital times a spin function. The use of these functions implies that electron correlation is neglected. The electron–electron repulsion is only included as an average effect.

### 2.3. Post-Ab Initio Methods

The third class of electronic structure methods, such as the Density Functional Theory (DFT), configuration interaction (CI) and Moller–Plesset Perturbation Theory (MP) methods, known as “*post-ab initio methods*” has recently been developed. These methods are attractive because they take the electron correlation into account. The DFT methods are the most widely used post-ab initio methods because they are thought as being the least expensive. The DFT methods use the electron density instead of the wavefunction. While the complexity of a wavefunction increases with the number of electrons, the electron density has the same number of variables. The DFT methods design functionals connecting the electron density with the energy.

### 2.4. Molecular Dynamics Method

The molecular dynamics methods combine energy calculations with the laws of classical mechanics. The simulation is performed numerically integrating Newton’s equation of motion over small time steps. Once the velocities are computed, new atom locations and the temperature of the assembly can be calculated.

### 2.5. Solvent Effect

All of the molecular modeling methods, molecular mechanics, semiempirical, ab initio, post-ab initio and molecular dynamics methods assume that the molecules are isolated from each other as in the gas phase. However, solvation plays a decisive role in determining the energetics of the reactions in aqueous media. Molecular modeling allows us also to compute the properties of the systems in solution.

In solutions, solvent molecules affect the properties of the solute molecules and the kinetics of the reactions. In aqueous media, solvent water affects the energetics of the solute species and also induces geometry relaxation for systems containing hydrogen-bonded complexes. However, previous results indicate that geometry changes have a negligible effect on the energy of the solute in water for both open and closed shell structures [42,43].

Solvation effects are generally modeled by polarizable continuum models (PCMs). In these methods, solvent is treated as a polarizable continuum rather than separate, individual molecules in order to reduce the computational cost. The solute is placed into a cavity within the solvent. The construction of the cavity is different in different PCM methods. In most cases, it is constructed as an assembly of atom-centered spheres, while the cavity surface is approximated by segments.

Among PCMs, the so-called conductor-like screening model COSMO is popular to use for aqueous media [42]. In COSMO, the solvent is treated as a dielectric continuum surrounding the solute molecule. Solute molecule forms a cavity with a similar shape. The charge distribution on the solute polarizes the dielectric medium. This effect causes the generation of screening charges on the cavity surface. Therefore, solvent is described by apparent polarization charges included in the solute Hamiltonian, so that it is possible to perform iterative procedures leading to self-consistence between the solute wavefunction and the solvent polarization. The COSMO method describes the dielectric continuum by means of apparent polarization charges distributed on the cavity surface, which are determined by imposing that the total electrostatic potential cancels out on the surface. This condition describes the solvation in polar liquids. Hence, COSMO can be accepted as a suitable method to be used in TiO_2_ photocatalysis.

## 3. Degradation Reaction Model

### 3.1. Active Species

In TiO_2_ photocatalysis studies, the reaction model used so far has been the reaction between the pollutant molecule and the •OH radical [44,45]. However, the governing role of active species leading to initial photocatalytic process is still a matter of controversy. The interfacial transfer of conduction band electrons to the adsorbed oxygen acting as primary electron acceptor has been accepted as the rate-determining step of the whole photocatalytic reaction. The most important primary chemical process is the formation of •OH radicals from adsorbed OH groups. These radicals either diffuse in solution or migrate on the surface. During migration, other species like H_2_O_2_ or peroxyl radicals are formed. However, •OH radicals are considered to be the principle reactive species responsible for the photocatalytic reaction.

**Figure 1:**
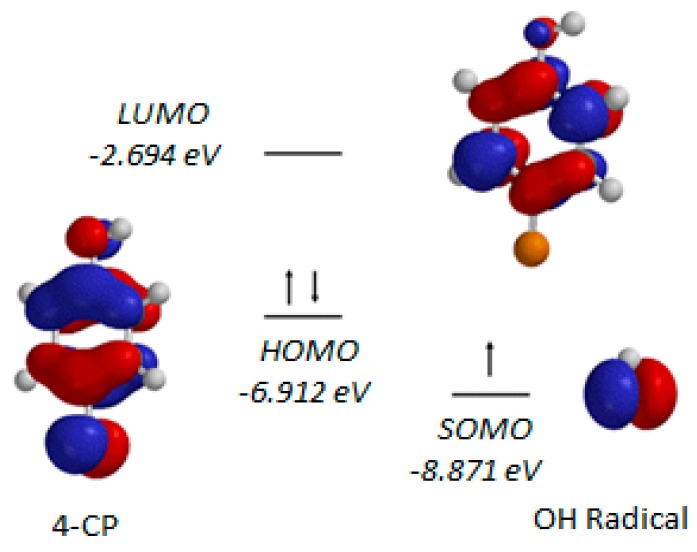
The frontier orbitals of 4-chlorophenol and the adsorbed •OH radical.

In order to compare the behavior of the two species, the frontier orbitals of the free and the adsorbed •OH radicals on TiO_2_ have been calculated by DFT/B3LYP/6-31G* method [46]. The results obtained indicate that the •OH radical is strongly bound to the TiO_2_ surface, the distance between the oxygen atom of the •OH radical and the surface titanium cation has been calculated to be 1.822 A. Moreover, it has been also found that the frontier-orbital energy of the •OH radical does not change much, the SOMO (singly occupied molecular orbital) of the adsorbed •OH radical has been calculated to be −8.871 eV, slightly higher than the energy of the free •OH, −8.953 eV. The frontier orbitals of 4-chlorophenol (4-CP) and the adsorbed •OH radical are presented in Figure 1. Therefore, it may be concluded that the photocatalytic degradation reactions of compounds in the presence of TiO_2_ may be based on hydroxyl radical chemistry. The most plausible reaction pathway for hydroxyl radical having a strong electrophilic character is a direct attack on one of the atoms of the pollutant molecule, generally the one with the highest electron density.

### 3.2. Reaction Center

Most of the organic pollutant molecules are aromatic in nature. •OH radicals react with aromatic molecules through addition to yield hydroxycyclohexadienyl type radicals which then form intermediates. Due to its highly electrophilic character, •OH radical has a very low-lying SOMO (singly occupied molecular orbital) the energy of which is −8.871 eV, while the energies of the HOMO (highest occupied molecular orbital) and LUMO (lowest unoccupied molecular orbital) of the aromatic molecules are around −5.4 and −2.7 eV respectively. Therefore, in the hydroxylation of the aromatic molecules, the SOMO of the •OH radical interacts with the HOMOs of the molecules, as displayed in Figure 1 for 4-chlorophenol. It may be concluded that the photocatalytic degradation reactions on TiO_2_ are orbital-controlled reactions.

It has been experimentally proven that •OH radicals attack aromatic molecules at the ring positions. Therefore, the intermediates and products of the degradation reactions of aromatic molecules depend upon the position of attack of the •OH radicals. There are several theoretical shortcut methods in the literature for the determination of the position of attack of the •OH radical in such reactions [47]. One of the most successful ones is the “frontier orbital theory (FMO)” which states that in electrophilic reactions, the point of attack is at the position of the greatest electron density in the HOMO of the aromatic molecule. The HOMO coefficients for 4-nitrophenol (4-NP) have been calculated by using a semiempirical PM3 (Parametric Model number 3) method to be 0.4 for the two -*ortho* positions and 0.1 for the two-*meta* positions with respect to the functional –OH group. These values indicate a high preference for the -*ortho* carbons and the most probable primary intermediate that forms in the photocatalytic degradation of 4-NP has been predicted to be 1,2-dihydroxy-4-nitro-cyclohexadienyl radical which then forms 4-nitrocatechol [48].

Another theory for the determination of the position of attack of the •OH radical to the aromatic molecule is the localization approach of Wheland [47]. According to this theory, the position of attack is determined by the energy of the intermediate. The most probable intermediate is the one with the lowest energy. The calculated total energies and the heats of formation of the hydroxylated radicals forming in the photocatalytic degradation reaction of 4-NP indicate that the attack is at the -*ortho* position in agreement with the FMO Theory [48].

## 4. Prediction of Degradability

Quantum mechanical methods are very useful tools for obtaining information about the degradabilities of the pollutant molecules. In fact, the degradation rate depends upon the electronic structure of the compound. However, there are relatively few correlations between the degradability and the molecular structure, reported in the literature [49,50,51]. In all of these equations, empirical parameters, such as 1-octanol/water partition coefficient, the Hammett constant, molecular refractivity and Brown constant have been used as the molecular properties. Therefore, the correlations obtained contain either experimental or approximate structural parameters. The use of molecular modeling techniques gives better relationships.

### 4.1. Electronic Molecular Properties

The energies of the frontier molecular orbitals are important quantum mechanical indices, the application of which in quantitative structure activity relations (QSAR) has gained considerable attention in the last decades. The energy of HOMO (E_HOMO_) is a measure of the ease of oxidation of the compound. On the other hand, the energy of the lowest unoccupied orbital (E_LUMO_) generally shows the reduction potency of the compound. The excitation energy, which is defined as the difference in energies of the HOMO and LUMO, reflects the electronic stability of the pollutant molecule.

In order to obtain a relationship predicting the degradabilities of monosubstituted anilines, San and Cinar [52] have used the semi-empirical PM3 method to calculate electronic properties of the compounds and correlated the logarithm of the experimental degradation rate constant (k) with the energies of the HOMOs, (E_HOMO_) and the sum of the electron densities (q) of the substituents. E_HOMO_ is a measure of the ease of oxidation of the compound while 1-octanol/water partition coefficient (K_OW_) is a measure of the distribution of the compound between TiO_2_ particles and the solution. The sum of the electron densities has been used in order to take into account the electrophilic character of the •OH radical. As a result, a linear relationship of the form

log k = Aq + BE_HOMO_ + C log K_OW_ + D (2)

has been obtained by using multiple regression. In this equation, A, B, C and D are the constants obtained by regression. The correlation coefficient r has been calculated to be 0.9937 with A = 0.02, B = 0.22, C = −0.27 and D = −0.32.

### 4.2. DFT Reactivity Descriptors

The best descriptors showing degradability can be obtained by the application of the Conceptual Density Functional Theory (DFT). According to the Conceptual DFT, the reactivity of a molecule depends upon its response to the perturbations caused by the attacking chemical species, in TiO_2_ photocatalysis •OH radicals. The typical perturbations for a chemical reaction are changes in external potential and the number of electrons N. The Conceptual DFT discusses reactions in terms of these changing properties. This approach leads to a series of reactivity descriptors, such as; the electronic chemical potential, hardness, softness and Fukui function. These descriptors then may be connected to different reactivity principals to be used in the determination of the regioselectivity and the reactivity of the compound under investigation.

There are mainly two classes of DFT descriptors; global and local descriptors. Perturbations due to changes in the number of electrons are defined as global descriptors and are related to overall molecular stability. Perturbations due to changes in external potential are called local descriptors and determine the site selectivity of a molecule for a specific reaction type.

Global hardness *η* is defined as the second derivative of the energy *E* with respect to the number of electrons N. It is equal to the reciprocal of global softness (*S*) [53,54]. Using the finite

(3) $$\eta =\frac{1}{2}{\left(\frac{\partial^{2}E}{\partial N^{2}}\right)}_{v\left(r\right)}=\frac{1}{2S}$$

difference approach together with Koopman’s theorem, hardness can be written in terms of the first ionization potential (*I*) and the electron affinity (*A*) of the molecule, whereas, in the frozen-core approximation, global hardness equals the gap between the frontier orbitals;

(4) $$\eta =\frac{I-A}{2}=\frac{E_{LUMO}-E_{HOMO}}{2}$$

Global hardness is a measure of the stability of the molecule. It is also a measure of the resistance of a chemical species to change its electronic configuration. Therefore, stable molecules are likely to be harder than less stable molecules and thus they have low reactivities. On the other hand; global softness is related with the polarizability of the molecule. Soft molecules have a high polarizability, which can allow a large deformation of the electron cloud. Soft molecules are more reactive, thus their degradation rates are faster than the hard molecules.

Generally, local properties are used in the determination of the reactivities of different sites of a molecule. Fukui function *f*(*r*) is the most important local DFT descriptor. It is defined as the mixed second derivative of the energy of the molecule with respect to the number of electrons and the external potential:

(5) $$f(r)=\left(\frac{\partial^{2}E}{\partial N.\partial v(r)}\right)={\left[\frac{\partial \mu }{\partial v(r)}\right]}_{N}={\left[\frac{\partial \rho (r)}{\partial N}\right]}_{v(r)}$$

Fukui function reflects the reactivity of a certain site of the molecule and it is the change in the electron density driven by a change in the number of electrons. The larger the value of the Fukui function, the higher the reactivity of that site. The fundamental equations defining the Fukui functions per atom *i* in a molecule are;

(6) $$f_{i}^{-}=\left[q_{i}(N)-q_{i}(N-1)\right]$$

(7) $$f_{i}^{o}=\left[f_{i}^{+}+f_{i}^{-}\right]/2$$

where *q_i_* is the electron population of atom *i* in the molecule. $f^{-}\left(r\right)$ is used when the system undergoes an electrophilic attack, whereas $f_{i}^{o}$ governs radical attack. The local softness s(r) provides intermolecular reactivity information about regioselectivity. It is related to the Fukui function through s(r)=Sf(r). This equation indicates that f(r) redistributes the global softness among different parts of the molecule.

San et al. [55] have calculated global and local DFT descriptors for monosubstituted phenols and examined correlations between the experimental degradation rate constants and the calculated DFT descriptors. However, the results indicate that the global DFT descriptors do not well describe the degradabilities of the phenol derivatives due to their different adsorptive capacities and characteristics of the substituents. The calculated local descriptors give better results. This finding indicates that the photocatalytic degradation reactions of phenol derivatives are orbital-controlled reactions rather than radical attack.

The use of “*softness-matching principle*” which is based on the HSAB principle has given the best descriptor for phenol derivatives studied. According to the HSAB principle, soft atoms react preferentially with other soft atoms and hard atoms with other hard atoms. However, the principle also indicates that the interaction between two chemical species will not necessarily occur through their softest atoms but through those whose softnesses are approximately equal. The reactions investigated are orbital-controlled reactions in which soft–soft interactions dominate. For these, reactions, ∆*s* between the reacting atoms must be as small as possible. Therefore, $\Delta s=s^{+}\left(O\right)-s^{-}\left(C\right)$ the difference between the local softnesses of the oxygen atom of the attacking •OH radical and of the carbon atom of the aromatic ring that undergoes the electrophilic attack has been calculated for each of the phenol molecules.

**Figure 2:**
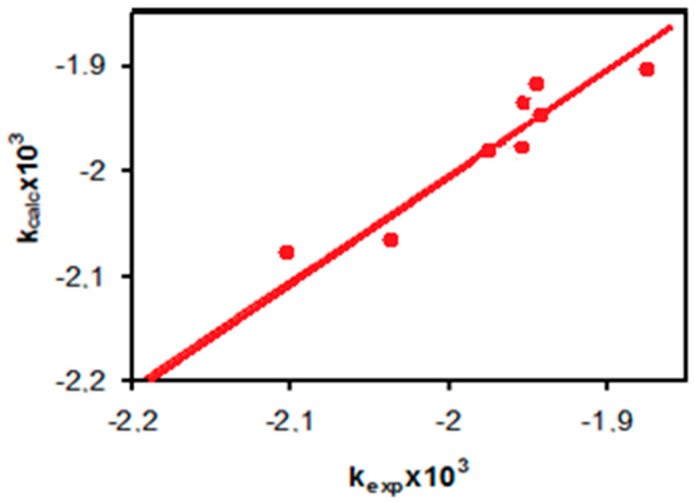
Observed vs. calculated rate constants for the photocatalytic degradation reactions of phenols.

Consequently, a simple linear equation giving logk in terms of ∆*s* and E_HOMO_ with a regression coefficient *r* = 0.9816 has been obtained. The experimental and calculated k values are presented in Figure 2. Therefore, it may be concluded that local DFT descriptors describe the reactivities of the phenol molecules in their photocatalytic degradation reactions better than the global ones.

## 5. Reaction Mechanisms

Molecular modeling methods can also be used to determine degradation reaction mechanisms. One way is to apply a short-cut method based on the use of DFT reactivity descriptors. However, the most reliable method is to apply Transition State Theory through quantum mechanical calculations.

### 5.1. Short-Cut Method

Short-cut method based on the use of DFT reactivity descriptors is computationally less demanding than transition state calculations. This method describes the preferred reaction energetics and thus kinetics in terms of the properties of the reactants in the ground state and is a successful tool to gain insight into how photocatalytic degradation reactions occur. The calculations based on reactivity indices are computationally less intensive but also less detailed because all information is obtained through study of the reactants only. Consequently, only information about the onset of the chemical reaction should be expected.

**Figure 3:**
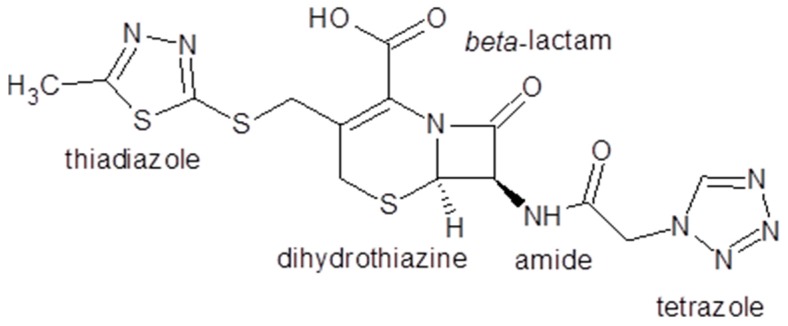
Cefazolin molecule.

In an earlier study [56], the degradation mechanism of cefazolin has been determined by using the DFT descriptors. Cefazolin is a semi-synthetic antibiotic. It belongs to the first generation of cephalosporines that are the most widely-used group of antibiotics and inhibits cell-wall biosynthesis in a manner similar to that of penicillins. As seen in Figure 3, cefazolin with molecular formula C_14_H_14_N_8_O_4_S_3_, consists of a fused β-lactam-∆^3^-dihydrothiazine two-ring system.

The reaction model used is the reaction between the cefazolin molecule and the photogenerated •OH radicals. Therefore, all the calculations have been based on hydroxyl radical chemistry. First the geometries of the reactants have been optimized and their electronic properties have been calculated. Then, the calculations have been repeated by adding and subtracting an electron from the reactant structures. Geometry optimizations of the reactants have been performed with the DFT method. The DFT calculations have been carried out using the hybrid B3LYP functional, which combines HF and Becke exchange terms with the Lee-Yang-Parr correlation functional in order to obtain an exact solution. 6-31G* has been used as the basis set.

Using the electron densities of the atoms in the optimized reactant structures Fukui functions have been calculated for each of the atoms in the molecule. Then, by using the highest *f* values local softnesses have been calculated. In order to determine the reaction centers and the reaction intermediates accordingly, softness-matching principle has been used. Therefore, for each of the atoms of the molecule $\Delta s^{o}=s^{o}(O)-s^{o}(X)$, the difference between local softnesses of the oxygen atom of the attacking •OH radical and the atom (*X*) of the cefazolin molecule that undergoes the radical attack has been calculated.

Then, the softnesses of the atoms have been compared with that of the •OH radical and the ones with softnesses being close to that of the •OH radical have been chosen. As a result, three main competing reaction pathways have been determined. The local softness *s*° and softness difference ∆*s°* values indicated that sulfur atoms on the thiadiazole ring are the prime targets of hydroxyl radical attack.

**Figure 4:**
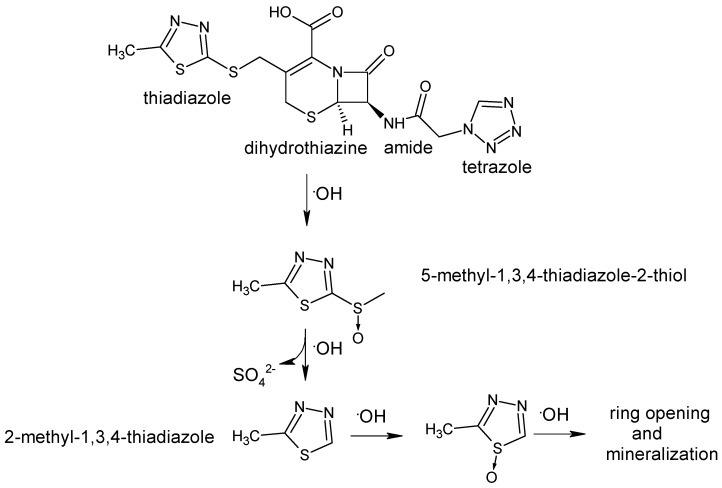
*Pathway I* for the photocatalytic degradation mechanism of cefazolin.

In *Pathway I*, upon the attack of the •OH radical, S–C bond cleavage occurs leading to the formation of 5-methyl-1,3,4-thiadiazole-2-thiol as seen in Figure 4. Oxidation of the other sulfur by •OH radicals precedes ring opening. Hydrogens on the dihydrothiazine are the two possible positions of •OH attack. Therefore, in the first step of *Pathway II*, •OH radical bonds to the carbon atom of the radical formed through H-abstraction. In the second step, lactonization occurs between hydroxyl and carboxy groups to yield a lactone as a by-product. The reactivity of the carbon on β-lactam ring is high according to its local softness value, in the third step, upon the attack of the •OH radical, β-lactam ring opens to give a β-lactam ring-opened lactone. The local softness and softness difference values of the atoms indicated that sulfur atom on the dihydrothiazine is another reactive site for •OH attack and it has been predicted that dihydrothiazine ring disappears when β-lactam ring is opened. In *Pathway III*, N–C bond cleavage occurs in the tetrazole part of the molecule. Successive •OH attacks results in ring opening and mineralization due to comparable reactivities of the nitrogen atoms on the tetrazole ring. All the predicted intermediates have been confirmed through FTIR, HPLC and UV-vis analyses.

### 5.2. Transition State Calculations

In order to determine the most plausible reaction path and the product distributions for the photocatalytic degradation reactions of organic pollutants the best way is to use Transition State Theory. For transition state calculations, the first thing to do is to carry out a conformer search for the reactants and the products to determine the most stable structures. Then, geometry optimizations of the reactants and the transition state complexes are performed by quantum mechanical methods. Zero-point corrections are made and the thermodynamic properties of all the species involved in the reactions are calculated.

Vibrational frequencies are calculated for the determination of the reactant and product structures as stationary points and true minima on the potential energy surfaces. All possible stationary geometries located as minima are generated by free rotation around single bonds. The forming C–O bonds in the OH-addition paths and the H–O bonds in the H-abstraction paths are chosen as the reaction coordinates in the determination of the transition states. Each transition state is characterized with only one negative eigenvalue in its force constant matrix.

Product distributions are determined by calculating the rate constant for each of the possible reaction paths by using the Transition State Theory. The classical rate constant k in the Transition State Theory is given by Equation (8);

(8) $$k=\frac{k_{B}T}{h}\frac{q_{TS}}{q_{R}.q_{OH}}e^{-E_{a}/RT}$$

where k_B_ is Boltzmann’s factor, T is temperature, h is Planck’s constant and q’s are molecular partition functions for the transition state complex (TS) and the reactant species, (R) and •OH and E_a_ is the activation energy. Each of the molecular partition functions is the product of the translational, rotational, vibrational and electronic partition functions of the corresponding species. The equations used for computing partition functions are those given in standard texts on thermodynamics. In the computation of the translational partition function, the molecule is treated as a particle with mass equal to the molecular mass of the molecule confined in a three-dimensional box. Vibrational partition function is composed of the sum of the contributions from each vibrational mode. The bottom of the potential well of the molecule is used as the zero of energy. Rotational partition function depends upon the geometry of the molecule, which is determined in the optimization step of the calculations, while electronic partition function is composed of the sum of the contributions from each electronic energy level. Thus, it depends upon the energies of the orbitals and their degeneracies. The first and higher excited states are assumed to be inaccessible at any temperature since the first excitation energy is much greater than k_B_T.

In an earlier study [57], in order to determine the product distributions and also to develop a short-cut model for the photocatalytic degradation reactions of phenol derivatives, their reactions with the photogenerated •OH radicals have been modeled. Forty-three different reaction paths for the reactions of 11 phenol derivatives with the •OH radical have been determined by nature of the carbon atoms of the aromatic ring, the substituent and the functional –OH group. •OH radical additions to the aromatic rings yielding dihydroxycyclohexadienyl type radicals and direct H-atom abstraction from the phenolic functionalities have been modeled. For all the possible reaction routes, calculations of the geometric parameters, the electronic and thermodynamic properties of the reactants, the product radicals and the transition state complexes have been performed with the semi-empirical PM3 and DFT-COSMO methods successively. The molecular orbital calculations have been carried out by a self-consistent field SCF method using the restricted RHF or unrestricted UHF Hartree–Fock formalisms depending upon the multiplicity of each species. Single point energies were then refined by DFT calculations. Based on the results of the calculations, the rate constant k for each reaction path has been calculated by using the transition state theory for 300 K. The branching ratios and the product distributions of all the possible reaction paths have been calculated by dividing the corresponding rate constant of each reaction path by overall k taking the number of similar addition centers into account.

The results obtained have been compared with the available experimental data in order to assess the reliability of the proposed model. The values indicate that DFT/B3LYP/6-31G*//PM3 calculations underestimate the rate constants. The reason may be attributed to the use of the PM3 method which is wavefunction based. The use of higher basis sets could modify the results, but they are not affordable in terms of computational time and resources. However, the primary intermediates determined in this study are in perfect agreement with the experimental ones. Therefore, it may be concluded that the proposed theoretical model, even by the PM3 method can be used for the estimation of the rates and the product distributions for the photocatalytic degradation reactions of phenol derivatives or similar pollutant molecules in gas and aqueous media [48,58,59,60].

**Figure 5:**
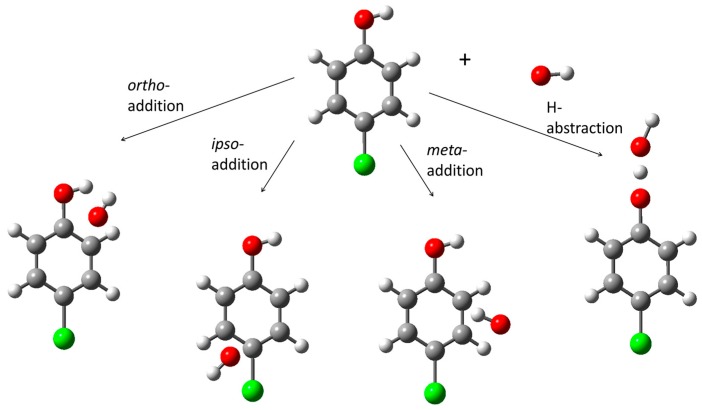
Possible reaction paths for 4-CP + •OH reaction.

With the aim of describing the mechanism of the photocatalytic degradation reaction of 4-chlorophenol (4-CP) in detail, the reaction between •OH radical and 4-CP has been modeled by means of the DFT method for geometry optimizations in order to determine the identities and the relative concentrations of the primary intermediates [46]. The DFT calculations have been performed by the hybrid B3LYP functional by using 6-31G* basis set. As seen in Figure 5, four different possible reaction paths for the reaction of 4-CP with the •OH radical have been determined by nature of the carbon atoms of the aromatic ring and the functional –OH group. The first three of the reaction paths, *ortho*-addition, *meta*-addition and *ipso*-addition are OH-addition reactions, which yield dihydroxy-chlorocyclohexadienyl type radicals. The fourth reaction path, H-abstraction is hydrogen abstraction from the functional –OH group producing 4-chlorophenoxyl radical and a water molecule. The unexpected result obtained in this study is that the energies of the transition states, optimized with the DFT/B3LYP/6-31G* method are lower than the energies of the reactants, 4-CP and the •OH radicals. This finding indicates that in all the reaction paths, pre-reactive complexes which lower the energy barriers are formed before the formation of the transition state complexes. Therefore, pre-reactive complexes (PCs) have been located on the potential energy surfaces. Similar results have been obtained for the degradation reactions of nitrobenzene and toluene [61,62].

The product distribution obtained shows that the major primary intermediate that is formed in the photocatalytic degradation of 4-CP is 1,2-dihydroxy-4-chlorocyclohexadienyl radical which then forms 4-chlorocatechol. The reaction also yields 1,4-dihydroxy-4-chlorohexadienyl radical through *ipso*-addition to the aromatic ring. As the chlorine substituent is then released due to the presence of water molecules and steric effects, this radical is converted to hydroquinone. The results obtained indicate that the major intermediates of the 4-CP + •OH reaction are 4-chlorocatechol (4-CC) and hydroquinone (HQ) with [4-CC] > [HQ], confirming the experimental findings of earlier studies reported in the literature [39].

In an earlier study, a combination of experimental and quantum mechanical methods has been used for the reaction of toluene with •OH radical in both gas and aqueous media in order to determine the most probable reaction path and the product distribution [62]. The obtained experimental and theoretical results are in perfect agreement. Both of them indicate that the dominant reaction path is the *ortho*-addition yielding *ortho*-cresol. Quantum mechanical results show that the primary intermediate is 1-hydroxy-2-methylcyclohexadienyl radical which then forms *ortho*-cresol through abstraction of the redundant ring hydrogen by molecular oxygen.

## 6. TiO_2_ Surfaces

Molecular modeling techniques can also be used to examine TiO_2_ surfaces either modified or doped. Localized and delocalized modeling methods are the two main modeling techniques to be used in the quantum mechanical studies of crystalline solids and surfaces. In delocalized modeling technique, it is assumed that the presence of translational symmetry in crystalline solids leads to periodic functions as solutions of the Schrödinger equation. The simplest periodic functions are known as plane waves. Periodic models are advantageous, because they have no surfaces, but they need exceptionally large repeating units that increase the computational cost. In localized modeling technique, finite clusters are modeled by using molecular orbitals instead of plane waves. In contrast to periodic models, cluster models use small representative portions of the crystal that can be treated with molecular quantum mechanical methods. Plane waves are delocalized and do not refer to a particular site in the crystal lattice. However, surface modifiers, dopants and defects are localized, thus their description by cluster models is favorable over periodic models. There is one problem in the use of cluster models: they have more surface area than the real crystal. Free clusters have borders such as corners, surfaces and edges that are not present in the bulk of the crystal. Therefore, the surface area should be reduced by saturating the unsaturated atoms at the surface by other type of atoms or groups that are similar to the ones in the environment of the crystal.

### 6.1. Doped TiO_2_ Surfaces

The anatase phase is the most used TiO_2_ photocatalyst. Among the low-index planes, (101), (100) and (001) lattice planes present on the surface of anatase, (001) surface is the most stable surface with a high photocatalytic activity [63,64]. Therefore, to determine the electronic properties of the doped or surface modified TiO_2_ surfaces, the non-defective undoped anatase (001) surface is modeled first with finite, neutral and stoichiometric cluster models. The reason for using neutral clusters is to avoid associating formal charges with the cluster. The bare TiO_2_ cluster models can be constructed by using the structure of the anatase unit cell. The unit cell for anatase has a tetragonal structure with the bulk lattice constants *a* = *b* = 3.78 A and *c* = 9.51 A [65]. The building stone is a slightly distorted TiO_6_ octahedron, with the oxygens at the corners. Each octahedron is in contact with eight neighbors, four sharing an edge and four a corner. Ti^4+^ cations are coordinated to six O^2−^ anions and the oxygen atoms are coordinated to three titanium atoms. The small cluster models can be enlarged by extending the lattice vectors to construct larger models, supercells. However, the anatase surface is Lewis acidic due to the presence of adsorbed water molecules [66]. Water adsorption on anatase occurs mostly by dissociative adsorption. Therefore, in the cluster models, to saturate the free valence at the surface and also to keep the coordination of the surface atoms the same as that in the bulk, the unsaturated oxygens are terminated with hydrogens and titaniums with OH groups.

In an earlier study, the bare (001) surface of anatase has been modeled by two different sized cluster models and the electronic properties of the cluster surfaces have been calculated by quantum chemical methods [63]. All the calculations have been performed using the DFT/B3LYP method within the GAUSSIAN 03 package [67] because it takes electron correlation into account. The double-zeta LanL2DZ basis set has been used in order to take relativistic effects into account. The cluster geometry has been kept frozen throughout all the calculations, but the terminal hydrogens and the OH groups are relaxed. Electronic structure calculations indicate that the upper states of the valence band (VB) are dominated by O 2p orbitals, while the bottom of the conduction band (CB) is mainly due to Ti 3d orbitals. The DFT/B3LYP calculations for such clusters generally underestimate band-gap energies, due to the well-known shortcoming of the exchange–correlation potential used within the framework of DFT [68,69]. However, calculated band-gap energies for small clusters do not reflect this effect, because the energy of the first excited state increases as the model size decreases. Band-gap energies for large clusters are corrected by using a scissors operator that displaces the empty and occupied bands relative to each other by a rigid shift to bring the minimum band-gap in line with experimental band-gap of anatase.

In order to determine the location and the bonding status of the dopants, doped anatase clusters are modeled. The doped models can be constructed by locating the dopant either substitutionally or interstitially depending upon the radius of the dopant into the bare anatase cluster. Then, site preference of the dopant on the surface is determined by changing the position of the dopant and calculating total energies of the doped clusters. The geometric parameters, the band edges, band-gap energies and Mulliken charge distributions of the surface atoms can be calculated for the doped model with the optimum dopant position.

**Figure 6:**
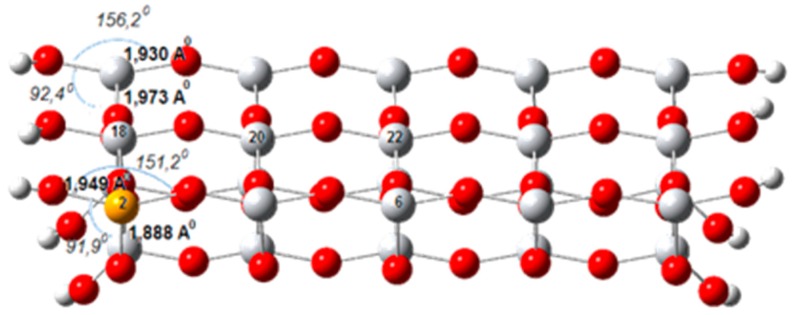
Optimized structure of the Se(IV)-doped TiO_2_ cluster model (grey, titanium; red, oxygen; orange, selenium; white, hydrogen).

Gurkan et al. [63] have examined selenium (IV)-doped TiO_2_ experimentally and theoretically. Only substitutional sites for Se (IV) ion have been analyzed. The doped models have been constructed by replacing one titanium atom by one selenium atom as seen in Figure 6. DFT/B3LYP/LanL2DZ calculations indicate that four-fold coordinated Ti site substitution is favored over five-fold coordinated Ti site substitution. Moreover, the results show that doping with Se (IV) does not cause a significant change in the positions of the band edges; instead, it introduces three empty mid-gap levels into the band-gap. The calculated coefficients of the wavefunctions indicate that they are mainly originating from the Se 3p orbitals. These energy levels are not donor states because they are not populated by electrons, but they are allowed energy states. These levels separate the band-gap of the Se(IV)-doped TiO_2_ into two parts; a wider lower gap and a significantly narrower upper gap. These intermediate energy levels offer additional steps for the absorption of low energy photons through the excitation of VB electrons to these intermediate energy levels, from where they can be excited again to the CB. The lower gap has been calculated to be 2.85 eV corresponding to a 435 nm photon. The result is consistent with the UV-DRS spectrum of the sample which shows two absorption thresholds. Therefore, it may be stated that the lower gap is responsible for the absorption in the first region of the spectrum between 420 and 580 nm, while the second region between 580 and 650 nm corresponds to the excitation of electrons from mid-gap levels to the CB.

### 6.2. Surface Modifiers

Surface modifiers enhance the surface coverage of pollutant molecules on TiO_2_, inhibit e^−^/h^+^ recombination process by separating the charge pairs and expand the wavelength response range. Benzoic acid, salicylic acid and ascorbic acid have been used as surface modifiers to increase the photocatalytic activity of TiO_2_ [70,71]. The experimental results indicate that these compounds modify the surface of the particles through the formation of π–π donor–acceptor complexes [72]. Salicylic acid SA has been used as a modifier for TiO_2_ and it has been reported that the onset of absorption of the SA-modified TiO_2_ shifts to the red and the threshold of absorption is at 420 nm compared with 380 nm for the unmodified TiO_2_ [70]. Although it has been known that surface modification by aromatic carboxylic acids increases the photocatalytic activity of TiO_2_, the key question to be answered is the electronic nature of the surface and the role of the surface complexes that lead the absorption edge to be red-shifted.

**Figure 7:**
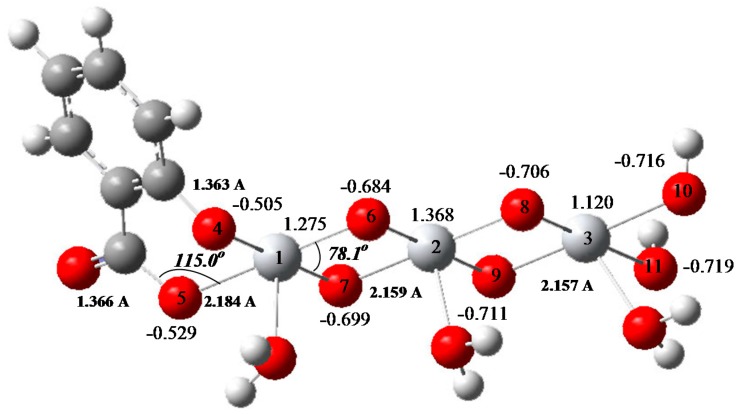
SA-modifed TiO_2_ cluster model (Grey, titanium; red, oxygen; white, hydrogen; black, carbon).

In order to investigate the effect of surface modification with salicylic acid SA, the non-defective anatase (001) surface modified with SA has been modeled with a finite, neutral, stoichiometric cluster model SA-Ti_3_O_11_H_8_ [73]. Almost 40% of the TiO_2_ surface consists of Ti atoms whose coordination is incomplete [74]. These atoms are four-fold coordinated to oxygen and have two unfilled orbitals. Therefore, they can accept two lone electron pairs from electron donors to complete the octahedral coordination [71]. In the SA-modified TiO_2_, the oxygen atoms of SA bond to these Ti cations, resulting in a chelate structure as seen in Figure 7. Both carboxyl and hydroxyl groups of SA are involved in binding. A bidentate binding of SA occurs through the two oxygen atoms yielding a six-membered ring structure which is the favorable configuration of the surface Ti atoms. As a result of surface modification, titanium(IV) salicylate charge-transfer complex is formed on TiO_2_ particles through the charge transfer reaction from the modifier (SA) to the conduction band of TiO_2_.

Electronic structure calculations indicate that conduction band minimum (CBM) shifts to a lower energy level in the SA-modified TiO_2_ with respect to the bare TiO_2_ due to the electron transfer from SA to the conduction band (CB) of TiO_2_. The formation of the bidentate charge transfer complex on TiO_2_ introduces additional electronic states into the band gap. The results indicate that the formation of the SA-bidentate complex on TiO_2_ particles causes an important reduction in the band gap of the photocatalyst. As a result of the study, 396 nm for the absorption threshold of the SA-TiO_2_ has been obtained which is in agreement with the experimental result reported in the literature [70].

The surface modification does not only reduce the band gap, but it also causes a change in the local charge distribution of the atoms on TiO_2_ surface. The Mulliken charge distribution of the atoms on the surface of the SA-TiO_2_ indicates that electron transfer occurs from SA to the conduction band of TiO_2_, causing the separation of the charge pairs on the surface into two phases. The holes on the complex can readily oxidize the adsorbed OH^−^ ions or H_2_O molecules and form •OH radicals. As the holes are on the bidentate complex and the electrons are on the TiO_2_ cluster, the electron–hole recombination process will be greatly inhibited, resulting in an increase in the photocatalytic degradation rates of organic contaminants. Similar results have been obtained for ascorbic acid modified TiO_2_ samples through quantum mechanical calculations [71].

## 7. Summary and Outlook

This review highlights the most important molecular modeling methods and applications in TiO_2_ photocatalysis. The relative advances in the synthesis and preparation of TiO_2_ photocatalysts have led to a huge range of novel materials with improved properties and surface phenomena. The photocatalytic degradation reactions of pollutants have gained considerable attention in the last decade due to an increased number of hazardous synthetic pollutants that are being consumed. Selected examples of the applications of molecular modeling methods to the problems of TiO_2_ photocatalysis have been explained.

Molecular modeling techniques are very important tools for TiO_2_ photocatalysis. They use quantum mechanical methods that allow us to study chemical phenomena by running calculations on computers rather than carrying out experiments. Quantum mechanical calculations appear promising as a means of describing the mechanisms and the product distributions of the photocatalytic degradation reactions of organic pollutants in both gas and aqueous phases. Since quantum mechanical methods utilize the principles of particle physics, their use may be extended to the design of new photocatalysts.

In TiO_2_ photocatalysis the primary process is the generation of •OH radicals from the adsorbed OH groups. •OH radicals are considered the principle reactive species responsible for the photocatalytic reaction. Therefore, photocatalytic degradation reactions of aromatic pollutants on TiO_2_ may be based on hydroxyl radical chemistry. Local DFT descriptors describe the reactivities of phenol derivatives and indicate that the photocatalytic degradation reactions are orbital-controlled reactions in which soft–soft interactions dominate. Product distributions may be predicted using even semiempirical quantum mechanical methods. However, DFT methods describe the reaction mechanisms better than the semiempirical ones. COSMO method is a suitable solvation model to be used to describe the reaction kinetics in aqueous media. Surface modification by aromatic carboxylic acids reduces the band gap of TiO_2_ and inhibits electron–hole recombination process by causing the separation of charges on the surface. Dopants reduce the band-gap either by introducing additional electronic energy levels into the band gap or contributing electrons to the VB of TiO_2_.

Novel TiO_2_ photocatalysts are visible-light active and they have higher photocatalytic activities than the standard anatase phase. The key question to be answered is how doping, codoping or surface modification achieve these phenomena. Molecular modeling methods can be used to obtain information about the degradabilities of the pollutants, to predict photocatalytic reaction mechanisms and evaluate the roles of impurities used to develop new photocatalysts. Many of these phenomena or the improved properties of the photocatalysts have not yet been explained in detail at the molecular level. I hope that the brief introduction of molecular modeling methods explained in this review, their applications in TiO_2_ photocatalysis and selected examples can provide readers necessary background and ideas to broaden the area of application of TiO_2_. There is no doubt that cooperative studies of computational chemists and experimentalists will lead to the fabrication of novel TiO_2_ photocatalysts and to the success of removing hazardous pollutants from air and water resources in the future.
